# TIdeS: A Comprehensive Framework for Accurate Open Reading Frame Identification and Classification in Eukaryotic Transcriptomes

**DOI:** 10.1093/gbe/evae252

**Published:** 2024-11-21

**Authors:** Xyrus X Maurer-Alcalá, Eunsoo Kim

**Affiliations:** Division of Invertebrate Zoology and Institute for Comparative Genomics, American Museum of Natural History, New York, NY, USA; Division of Invertebrate Zoology and Institute for Comparative Genomics, American Museum of Natural History, New York, NY, USA; Division of EcoScience, Ewha Womans University, Seoul, South Korea

**Keywords:** ORF prediction, machine learning, contamination, phylogenomics, biotic interactions

## Abstract

Studying fundamental aspects of eukaryotic biology through genetic information can face numerous challenges, including contamination and intricate biotic interactions, which are particularly pronounced when working with uncultured eukaryotes. However, existing tools for predicting open reading frames (ORFs) from transcriptomes are limited in these scenarios. Here we introduce Transcript Identification and Selection (TIdeS), a framework designed to address these nontrivial challenges associated with current ‘omics approaches. Using transcriptomes from 32 taxa, representing the breadth of eukaryotic diversity, TIdeS outperforms most conventional ORF-prediction methods (i.e. TransDecoder), identifying a greater proportion of complete and in-frame ORFs. Additionally, TIdeS accurately classifies ORFs using minimal input data, even in the presence of “heavy contamination”. This built-in flexibility extends to previously unexplored biological interactions, offering a robust single-stop solution for precise ORF predictions and subsequent decontamination. Beyond applications in phylogenomic-based studies, TIdeS provides a robust means to explore biotic interactions in eukaryotes (e.g. host–symbiont, prey–predator) and for reproducible dataset curation from transcriptomes and genomes.

**Significance**

Inferring open reading frames (ORFs) from eukaryotic transcriptomes can strongly impact the quality of downstream analyses, as poorly inferred and “contaminant” (e.g. prey organisms) ORFs can be challenging to discern. We present Transcript Identification and Selection (TIdeS) as a robust machine-learning framework that provides partial and full-length ORF predictions, outperforming popular tools and approaches for ORF prediction from eukaryotic transcriptomes. Additionally, we demonstrate TIdeS's ability to accurately discern ORFs from mixed samples, which are common in eukaryotic transcriptome sequencing projects (e.g. host vs. pathogen and predator vs. prey), with minimal human curation.

## Background

Generation of pure, large-scale genetic data from protists (i.e. eukaryotes outside animals, fungi, and plants) can be challenging, especially as high-quality, contamination-free molecular sequence data are a prerequisite for robust comparative evolutionary studies. For one, protist cultures often include co-occurring microbial species (Aponte et al. 2021), which may be difficult, or even impossible, to remove completely prior to sequencing due to symbiotic or prey–predator relationships. Additionally, given the labor-intensive nature of protist isolation and culturing, the vast majority of protists remain uncultured. This has, at least partially, contributed to the widespread adoption of cultivation-free, single-cell sequencing of genomes and transcriptomes, and has further expanded exploration of eukaryotic biodiversity and evolutionary biology (Lax et al. 2018; Treitli et al. 2019; Yan et al. 2019).

Most eukaryotes lack high-quality reference genome data, and given the relative ease and low cost of sequence data generation, de novo assembled transcriptomes tend to act as the sole reference datasets for a number of major eukaryotic lineages. These large assembled single (or few) cell derived transcriptomes have been transformative in shaping our understanding of the eukaryotic tree of life (Lax et al. 2018; Schön et al. 2021), evolution (Yan et al. 2019), and inter-organismal associations (e.g. symbioses; Boscaro et al. 2023). A major step in these analyses includes the prediction of open reading frames (ORFs) to identify putative protein-coding sequences that can be further analyzed. ORF calling represents its own unique challenges, in part given the high-representation of incomplete ORFs from assemblies using short-read technologies (Smith-Unna et al. 2016) and the identification of phylogenetically diverse lineages of eukaryotes where canonical stop codons have been reassigned to sense (Heaphy et al. 2016; Záhonová et al. 2016; Bachvaroff 2019).

Among the myriad of ORF-calling tools, TransDecoder (Haas et al. 2013) remains the “gold standard” for ORF calling and annotation. While numerous tools and approaches have been developed as alternatives to TransDecoder [e.g. CodAn (Nachtigall et al. 2021), GeneMarkS-T (Tang et al. 2015), and Prodigal (Hyatt et al. 2010)], these tools predominantly target a limited breadth of eukaryotic diversity (e.g. CodAn), often do not support alternative noncanonical genetic codes (i.e. reassigned stop to sense codons; CodAn and GeneMarkS-T), or were designed for prokaryotic ORF predictions (Prodigal). For example, CodAn provides pretrained models for fungi, plants, invertebrates, and vertebrates. While these taxonomic groups represent a substantial share of scientific study, they also represent a sliver of eukaryotic diversity. Neither CodAn nor GeneMark-ST support the majority of alternative genetic codes found just among the Ciliophora, where seven alternative genetic codes are present (Heaphy et al. 2016; Yan et al. 2019), making them ill suited, compared to TransDecoder, for working with eukaryotic taxa with alternative noncanonical genetic codes.

Accurate ORF predictions represent only a portion of the challenges working with transcriptome-based data from microbial eukaryotes. Established protist cultures often include one or more of co-cultured microbial species (Aponte et al. 2021). *Telonema*, for example, is a unicellular eukaryote that feeds on other photosynthetic or nonphotosynthetic unicellular protists; as such, *Telonema* cultures are not uniprotistan and their maintenance requires the addition of prey protist cells, such as the gliding small heterotrophic protist *Mantamonas* (Klaveness et al. 2005; personal observation). These issues are only compounded by the inability to fully dissociate protists from their own microbiomes (or host material), ultimately generating meta-transcriptomes/genomes (Boscaro et al. 2023). Several approaches are widely used, which vary from short-read classification using metagenomic approaches (e.g. Kraken2; Wood et al. 2019), to assessing assembled transcripts with “BLAST”-based approaches (e.g. BlobTools and “BLAST”-ing against NCBI's NR database; Camacho et al. 2009, Laetsch and Blaxter 2017) or with phylogenomic-based approaches (e.g. Cote-L’Heureux et al. 2022). Many studies tend to use a combination of approaches to aid in identification of transcripts and ORFs from target and nontarget taxa, as metagenomic and BLAST-based approaches tend to work best for prokaryotic contamination (although there is the potential for misattributing lateral gene transfer events to inter-domain lateral gene transfers). For many microbial eukaryotes, which lack strong representation across various public databases, phylogenomic-based curation is crucial for reducing the nontarget taxon contamination, relying on the generation and evaluation of hundreds to thousands of single-gene phylogenies (Lax et al. 2018; Cote-L’Heureux et al. 2022). Data curation can be time consuming, challenging to reproduce where “manual” curation is the primary approach, and possibly create unintended biases (McCarthy et al. 2023). Overall, this creates a large bottleneck in data processing as it often takes multiple rounds of curation, including phylogeny generation and curation before the bulk of contamination is removed and can lead to the abandonment and elimination of critical taxa from a given dataset. The challenges inherent in working with single-cell transcriptomic data do not inherently offset the benefits, but they can hamper the strength of biological insights garnered from those taxa.

Here, we developed *Transcript Identification and Selection* (TIdeS), a framework for de novo ORF identification and classification from clean to highly contaminated RNA-seq assemblies using support vector classifiers and sequence composition. From de novo transcriptome assemblies of 32 diverse eukaryotic taxa, we show that ORF classification by TIdeS accurately identifies and captures the majority of complete ORFs in the transcriptome assemblies. We validate TIdeS's ability to accurately disentangle and classify ORFs from complex samples representing several common biological interactions (e.g. food sources, co-cultured contaminants, and symbionts), which are common challenges with the widespread adoption of culture-independent, single-cell RNA-seq approaches. Overall, TIdeS is a “one-stop-shop” enabling quality transcriptome processing, accurate ORF prediction, and subsequent classification/decontamination with minimal training data and computational experience. TIdeS provides a useful tool for diverse applications, from large-scale phylogenomic, transcriptome-based studies on “young” gene families, and exploring on-going host–symbiont/pathogen interactions. TIdeS is provided for use at: github.com/xxmalcala/TIdeS.

## Results

### TIdeS Overview—Simple Preparation and Curation of ORFs from Transcriptomes

**Fig. 1.**
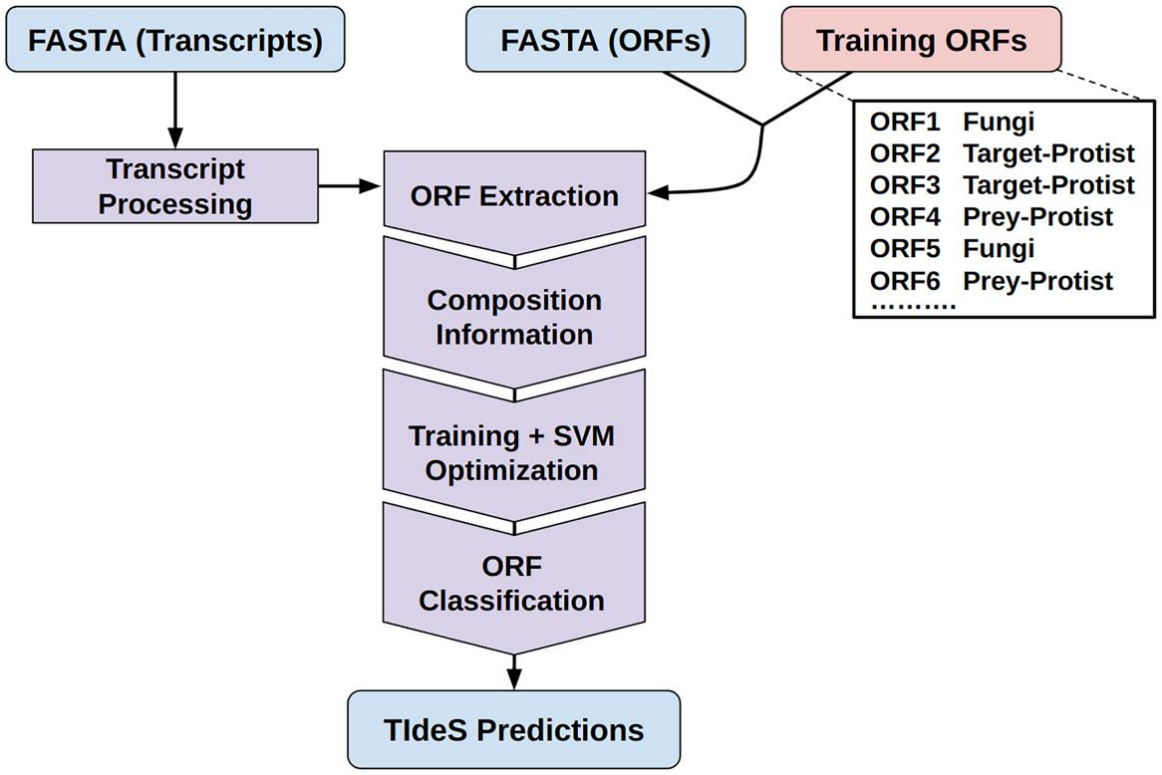
Overview of TIdeS workflow for ORF-calling and ORF-classification. For ORF-calling, only a FASTA file of transcripts is required, and can be *optionally* processed to remove short transcripts, rRNA by-catch and redundant isoforms. Afterwards, TIdeS extracts all ORFs (partial and/or complete). For ORF classification, a FASTA file of predicted ORFs and a table of user-defined ORFs need to be provided. Composition (k-mer-based) for all ORFs are extracted, with the data for training ORFs passed to the SVM for training and hyperparameter optimization. Finally, TIdeS classifies the query ORFs and generates its predictions, including a trained model, which can be provided as an optional input.

The initial processing for many transcriptomes following de novo assemblies includes identification and removal of redundant isoforms, rRNA bycatch, and nontarget taxon contamination. These steps are nontrivial and can substantially impact ORF prediction and downstream analyses (Van Vlierberghe et al. 2021). To address these challenges, TIdeS can be deployed in two general approaches: (i) transcriptome processing and ORF prediction and (ii) ORF classification and “decontamination” (Fig. 1). TIdeS handles the initial transcriptome processing and ORF prediction in two stages. The first stage includes removal of rRNA bycatch, followed by redundant isoform clustering. We have included an optional step to enrich for eukaryotic transcripts, using the metagenomic classifier, Kraken 2 (Wood et al. 2019), although we note that this is a nonexhaustive approach. The second stage includes the identification of all putative ORFs (pORFs) from the processed transcripts and generation of reference ORFs through a minimal reference-based orthology search using DIAMOND (Buchfink et al. 2021). Sequence composition metrics are then used to train a support vector classifier (SVC), which classifies the pool of pORFs, identifying the best “scoring” ORF as the optimal representative for a given transcript.

With a FASTA file of pORFs, TIdeS also supports their classification using either a small training dataset of user-classified pORFs from the same FASTA file, or from a previously trained classification model (Fig. 1). These pORFs can either be initially classified by their composition, quick Kraken2 assessments for noneukaryotic sequences, or through initial phylogenomic analyses. We provide a utility to calculate and visualize the relationship between the GC content of 1st/2nd codon positions (GC12) and the GC content of codon 3rd positions (GC3) that can be used to identify putative distinct “clusters” of sequences for classification. Overall, a combination of composition-based selection and user-inferred classes are recommended. Subsequent training and classification are similar to TIdeS's ORF prediction and assessment, using an SVC to efficiently classify ORFs into user-defined classes.

### TIdeS Produces More Quality ORF Predictions

Among the initial steps for many phylogenomic-based studies following assembly, involves the identification of ORFs. While numerous tools exist to tackle this crucial step, including GeneMarkS-T (Tang et al. 2015) and Prodigal (a prokaryotic gene prediction tool; Hyatt et al. 2010), few have become as well adopted as TransDecoder for RNA-seq based datasets. To assess ORF prediction with TIdeS, we predicted putative ORFs (pORFS) from a diverse breadth of eukaryotic taxa, using several common approaches: both the partial and complete ORF predictions from the TransDecoder, GeneMarkS-T, and Prodigal pipelines. We also evaluated the longest pORF *per* transcript (“longest-ORF”) as a common approach to ORF prediction. For each tool and approach, all partial and complete (i.e. possessing start and stop codons) pORFs were aligned against their respective proteomes using DIAMOND (Buchfink et al. 2021) to assess reading frames and completeness. We chose to evaluate the proportion of “useful” pORF predictions, where ≥98% of a given pORF's length is aligned across ≥66% of its reference protein's length, which we view as being most useful for a variety of downstream analyses (e.g. phylogenomics, gene ontology, etc.).

**Fig. 2.**
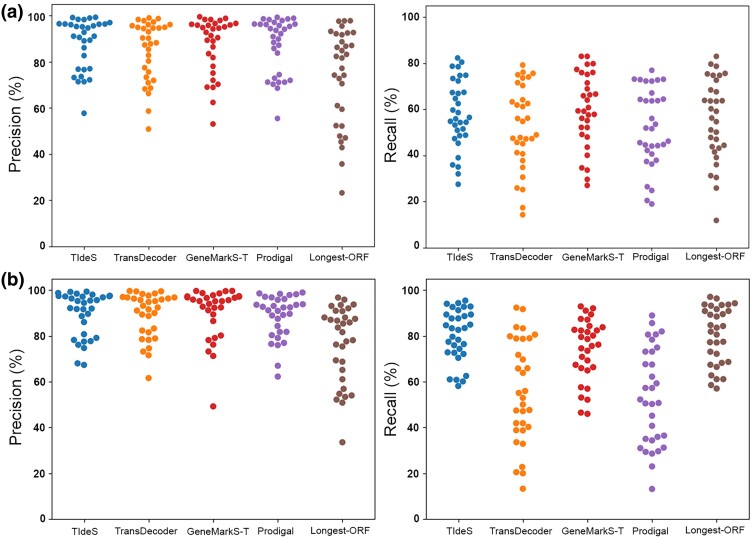
Comparisons of ORF prediction precision and recall for TIdeS and widely used ORF prediction tools and approaches. a) Total ORF prediction precision is comparable across the tools tested, whereas prediction recall by TIdeS and GeneMarkS-T greatly outperforms other approaches. b) TIdeS predicts complete ORFs, for evolutionarily diverse eukaryotic taxa, with greater precision and recall than common prediction tools and approaches.

Using default options, each approach showed similar levels of precision (proportion of ORFs in the correct reading frame (CRF) out of all predictions; Fig. 2a; Table 1), with TIdeS performing the best (88.0%), and GeneMarkS-T and Prodigal being near similar (87.2% and 87.3%, respectively), with TransDecoder exhibiting the lowest precision (85.0%) of the tools tested. We do note that the longest-ORF calling consistently performed the worst across the 32 taxa evaluated (74.0% of alignable pORFs in the CRF). Among the approaches deployed, the overall recall (proportion of all “useful” pORFs) was greatest in GeneMarkS-T (∼59.7%), followed by TIdeS (∼57.9%), and with TransDecoder and Prodigal performing similarly (52.3% and 52.1%) (Fig. 2a; Table 1). We note that this does include some redundancy in the pORFs (i.e. near identical isoforms). However, both TIdeS and GeneMarkS-T recover pORFs from a similar number of unique proteins (4,982 and 4,972, respectively), which is greater than TransDecoder and Prodigal (4,550 and 4,461, respectively). Overall, both TIdeS and GeneMarkS-T perform similarly in capturing “useful” ORF predictions, encompassing a greater protein diversity with improved precision and recall over TransDecoder and Prodigal.

**Table 1:** TIdeS performs comparably in total ORF-calling performance compared to common tools and approaches

Approach	Total ORFs	Unique loci	Average precision	Median precision	Average recall	Median recall	Average F1-score	Median F1-score
TIdeS	16,515	**4,982**	**0.880** ± **0.110**	0.920	0.579 ± 0.147	0.562	0.690 ± 0.132	0.692
TransDecoder	18,177	4,550	0.850 ± 0.131	0.892	0.523 ± 0.181	0.519	0.634 ± 0.172	0.634
GeneMarkS-T^a^	18,426	4,972	0.872 ± 0.125	0.928	**0.597** ± **0.160**	**0**.**593**	**0.698** ± **0.145**	**0**.**724**
Prodigal	18,402	4,461	0.873 ± 0.121	**0**.**929**	0.521 ± 0.169	0.517	0.639 ± 0.159	0.624
Longest ORF	**18,856**	4,870	0.740 ± 0.209	0.820	0.556 ± 0.178	0.577	0.625 ± 0.180	0.565

Bold values indicate the “best” performing approach for that metric.

^**a**^Data from Tetrahymena thermophila is excluded as nonstandard genetic codes are unsupported.

A major ORF prediction tools is to identify the greatest proportion of full-length ORFs in the CRF. To explore this, we further evaluated each approach's ability to capture full-length ORFs, which we define as those pORFs possessing start and stop codons whose alignments to reference proteins cover ≥98% of both the pORF and reference protein's lengths. These full-length ORFs comprise substantially different proportions of the pool of pORFs across the tools tested, comprising 34.1% of TransDecoder pORFs, 42.4% of GeneMarkS-T pORFs, only ∼29.9% of Prodigal pORFs. For these analyses, the TIdeS and longest-ORF approaches excluded evaluating partial ORFs, by default. From our set of 32 diverse eukaryotes, TIdeS and the longest-ORF approach possessed the greatest recall (proportion of all in-frame full-length ORFs), capturing ∼80.2% of all full-length ORFs, respectively, followed by GeneMarkS-T (73.9%), TransDecoder (55.8%), and Prodigal (53.3%) (Fig. 2b; Table 2). As with the prediction of “useful” pORFs, overall ORF-prediction precision was similar across tools (88.5% to 90.2%), whereas the longest-ORF approach exhibited the lowest precision (76.5%) (Fig. 2b; Table 2). However, the longest-ORF approach identified pORFs from the greatest proportion of the respective proteomes on average (3,754 unique loci), whereas Prodigal captured the smallest proportion from the eukaryotic proteomes (2,610 loci; Table 2). Among the tools tested, TIdeS identified full-length pORFs a greater proportion of the proteome for 31/32 taxa compared to both TransDecoder and Prodigal, and 29/31 taxa compared to GeneMarkS-T (note that *Tetrahymena* is excluded given GeneMarkS-T's inability to use nonstandard genetic codes; supplementary table S2, Supplementary Material online). Overall, TIdeS either performs similarly or outperforms common approaches and tools for calling ORFs from among a broad diversity of eukaryotes (Fig. 2).

**Table 2:** TIdeS predicts full-length ORFs with greater accuracy than common tools and approaches

Approach	Complete ORFs	Unique loci	Average precision	Median precision	Average recall	Median recall	Average F1-score	Median F1-score
TIdeS	10,961	3,689	0.895 ± 0.105	0.944	**0.802 ± 0.111**	**0**.**829**	**0.840** ± **0.099**	**0**.**829**
TransDecoder	6,193	2,789	0.893 ± 0.099	0.928	0.558 ± 0.224	0.540	0.663 ± 0.192	0.661
GeneMarkS-T^a^	7,819	3,347	**0.902** ± **0.113**	**0**.**951**	0.739 ± 0.135	0.762	0.804 ± 0.108	0.809
Prodigal	5,503	2,610	0.885 ± 0.094	0.918	0.533 ± 0.211	0.515	0.644 ± 0.184	0.659
Longest ORF	**12,641**	**3,754**	0.765 ± 0.167	0.823	**0.802** ± **0.126**	0.827	0.772 ± 0.130	0.772

Bold values indicate the “best” performing approach for that metric.

^**a**^Data from *Tetrahymena thermophila* is excluded as nonstandard genetic codes are unsupported.

### Limited Training Data are Needed for Accurate ORF Classification

**Fig. 3.**
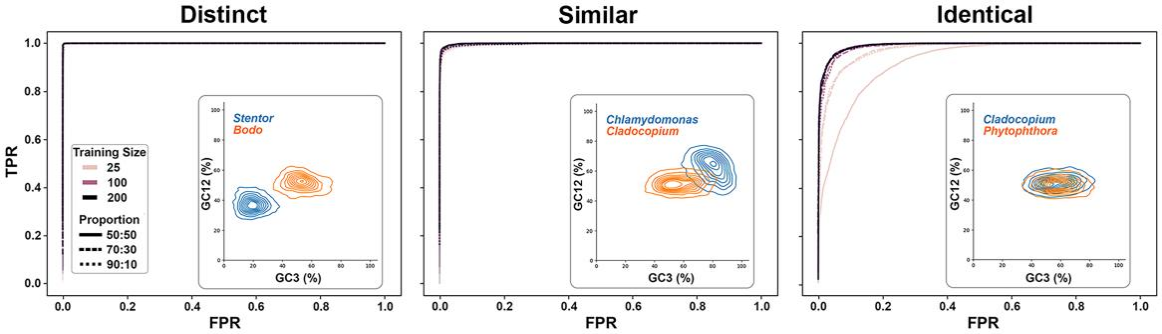
Limited training data are needed for accurate sequence classification. Across different scenarios of ORF composition (distinct, similar, and identical) and proportions of taxon representation, TIdeS can accurately classify ORFs with minimal training data. In scenarios where sequence composition is near identical, larger training datasets dramatically impact sequence classification, especially when taxon representation is near equal (i.e. 50:50). ORF composition is shown in each facet. %GC12: GC-content at codon 1st/2nd positions; %GC3: GC-content at codon 3rd positions.

Balancing training set size and overtraining is a nontrivial process that can dramatically impact predictive power. To determine the scale of training data necessary for TIdeS to accurately classify sequences, we pooled CDSs from binary sets of taxa with varying degrees of sequence representation, mimicking “contamination,” from equal to highly imbalanced proportions of sequences. Additionally, we aimed to include varying degrees of composition similarity across the samples to better mimic publicly available datasets that have perceptible contamination, ranging from easily separable to no perceptible differences in the relationships between GC12 and GC3, which we refer to as “distinct,” “dissimilar,” and “identical” (Fig. 3). Cases where the “contamination” is either “distinct” or “dissimilar” require only a limited set of, with 25 randomly picked sequences from each taxon (i.e. the minimum training size for TIdeS) was sufficient to accurately classify sequences to their respective taxa (Fig. 3). Imbalances in taxon representation (e.g. 50:50 to 90:10) show little impact on classification accuracy, although we note that when the imbalance is ∼90:10, the classification accuracy often approximates chance. In more “identical” datasets, TIdeS performance increases substantially with increasingly large training sets (e.g. 100 or more sequences from each representative; Fig. 3; supplementary table S3, Supplementary Material online).

### TIdeS Supports Accurate Multiclass Classifications

**Fig. 4.**
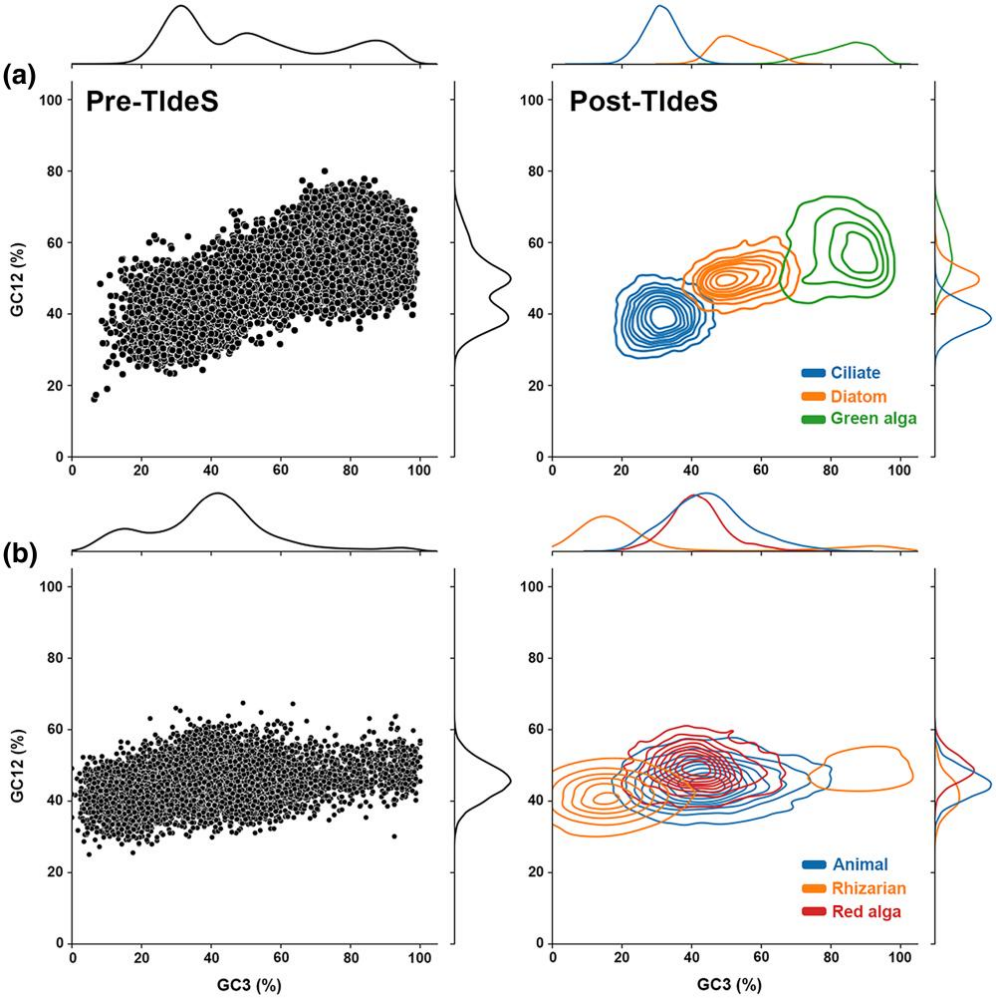
TIdeS can accurately classify ORFs in multitaxon scenarios. Left, sequence composition plots for the unclassified predicted ORFs from a) an in silico contaminated transcriptome of a large predatory ciliate (*Stentor coeruleus*), diatom (*Phaeodactylum tricornutum*), and green alga (*Chlamydomonas reinhardtii*) and b) a transcriptome of a red alga (*Lithophyllum stictiforme*) heavily contaminated with an unidentified metazoan and a rhizarian. Right, sequence composition plot following classification with TIdeS, using minimal training data based on ORF composition from the initial Pre-TIdeS plot. %GC12: GC-content at codon 1st/2nd positions; %GC3: GC-content at codon 3rd positions.

Many datasets generated by RNA-seq approaches are often better viewed as “metatranscriptomic,” as conditions are rarely free of common biotic interactions, including predator–prey and host–pathogen, despite best efforts to reduce “contamination” from nontarget taxa. With the inherent amplification bias of contaminant material from single-cell approaches (e.g. microbiome), we created an in silico microbiome, comprising the large predatory ciliate *Stentor coeruleus*, its preferred green algal prey *Chlamydomonas reinhardtii*, and the diatom *Phaeodactylum tricornutum*. Raw reads were pooled (*Stentor:* 63.8%, *Chlamydomonas*: 11.4%, and *Phaeodactylum*: 24.8% of all reads) prior to assembly to best mimic a “true” microbiome. We assessed the composition of the pORFs, following assembly and ORF prediction with TIdeS, identifying three putative clusters of sequences to classify. Random sets of 100 sequences were categorized into three clusters solely based on composition (see Materials and Methods). Despite solely relying on composition as a means to identify putative clusters of sequences, an arguably quick and “dirty” approach, TIdeS accurately classified the bulk of the dataset (Fig. 4a; Macro-F1: 0.947; supplementary table S4, Supplementary Material online). Discerning between *Chlamydomonas* and *Phaeodactylum* was the largest hurdle challenging TIdeS, likely due to greater overlap in their ORF compositions (Fig. 4a). Despite this limitation, TIdeS accurately classified 29,664/31,136 (95.3%) of sequences solely based on clusters specified by their compositions.

We also explored TIdeS's classification ability on a transcriptomic dataset of the red alga *Lithophyllum stictiforme*, which possessed strong contamination from metazoan and rhizarian taxa. An initial training dataset was created with the PhyloToL pipeline (Cerón-Romero et al. 2019) and subsequent tree-walking to rapidly assess sister-relationships for *L. stictiforme* sequences in 400 gene families, randomly selecting 100 ORFs for each dominant signal (i.e. red alga, metazoan, and rhizarian). With this small training sequence selection, we classified the remaining ORFs from a set of diagnostic single-gene phylogenies. TIdeS was able to accurately classify the bulk of sequences from *L. stictiforme* (F1-score: 0.917; Fig. 4b; supplementary table S6, Supplementary Material online), despite evidence for substantial ORF composition overlap between the red alga and its metazoan contaminant. We also compared these classifications with outputs from eggNOG-mapper and its taxon-rich database. Notably, while eggNOG-mapper was able to distinguish metazoan ORFs, this approach either reported numerous misclassifications (e.g. identification of rhizarian sequences as fungal in origin) or simply failed to distinctly classify sequences, which was pronounced for red-algal sequences. These issues dramatically impacted eggNOG-mapper's overall classification accuracy (F1-score: 0.508; supplementary table S6, Supplementary Material online). Despite eggNOG's high taxon representation, the lack of representative rhizarian and red-algal taxa is at least partly attributable to its poor classification ability for this dataset.

### Sequence Classification Opens up Opportunities for Other Biological Questions

Despite the best efforts of researchers, dissociating the target organism from their own microbiomes or other associated taxa (e.g. host, prey, or symbionts) often cannot be done prior to nucleic acid extraction and subsequent sequencing. This is particularly true for many microbial eukaryotes, where single-cell ‘omics techniques (e.g. single-cell RNA-seq and DNA-seq) are increasingly common and crucial. Mechanistic (e.g. size separation of cells using of membrane filters or by centrifugation at different g forces), physiological (e.g. starvation), or chemical (e.g. antibiotic regimes) approaches can be applied to reduce the nontarget abundance, but typically are unable to remove all nontarget taxa. Further, these treatments could impact the quality of the data and lead to altered outcomes, including impacting scRNA-seq amplification efficacy and putting cells in states of unaccounted physiological stress. This is particularly problematic when quality reference genomes are lacking and when assessing the association/infections is the experimental intent and goal. Given TIdeS's strong performance in simulated contamination scenarios, we sought to assess its performance using publicly available datasets representing different sets of unique challenges and potential implications, which we sort into two broad categories: contaminated sequencing samples and organismal associations. For all our datasets, we initially processed the de novo assembled transcriptomes with PhyloToL (Cerón-Romero et al. 2019) to generate a forest of 7,662 single-gene trees, of which 400 were used as a “diagnostic” set to ascertain putative contaminant/organismal associations. The remaining 7,262 single-gene phylogenies serve to score TIdeS's performance (e.g. how accurate are the classifications of target-taxon and nontarget sequences?).

### Classification of Sequences in Highly Contaminated Datasets

**Fig. 5.**
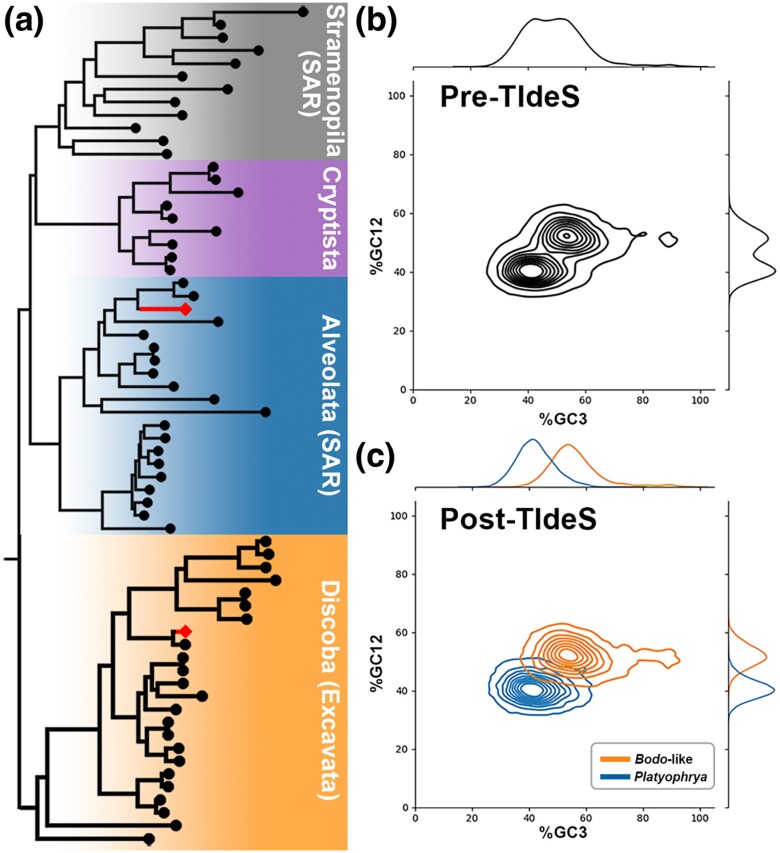
TIdeS accurately classifies ciliate (*Platyophrya*) and contaminant (*Bodo*-like flagellate) sequences with phylogenomic-inferred training data. a) Exemplar single-gene phylogeny shows *Platyophrya* protein sequences (diamonds) nestled among other ciliates (Alveolata) and unexpectedly among kinetoplastids (Discoba) implying a contaminated dataset. b) *Platyophrya*'s composition “fingerprint” suggests strong, but not easily delineated, contamination. c) TIdeS accurately classifies *Platyophrya* sequences from 50 training sequences, enabling easy phylogenomic identification of the contaminant data (*Bodo*-like nanoflagellate). b and c) %GC12: GC-content at codon 1st/2nd positions; %GC3: GC-content at codon 3rd positions.

When working with protist sequence data derived from isolated cultures or environmental material, small-sized protists and prokaryotes are common sources of sequence data “contamination”. From prior work and our de novo transcriptome assembly of the ciliate *Platyophrya macrostoma* (MMETSP0127; SRX551312), we identified a suitable major nonciliate signal from the 400 diagnostic gene trees, along with a composition “fingerprint” indicative of at least two taxa/sources (Fig. 5). From the diagnostic set of trees, the nonciliate source was predominantly associating with kinetoplastids (Discoba), which include many heterotrophic nano-flagellates (Adl et al. 2019). Using a small selection (100 training sequences each from *P. macrostoma* and “contaminant”) of the classified sequences from the diagnostic set of single-gene phylogenies, we were able to accurately classify sequences from *P. macrostoma* (F1-score: 0.987; Fig. 5; Table 3) and further identify a large number of contaminant ORFs (>18,000), predominantly belonging to a close relative of the nanoflagellate *Bodo saltans*. Using eggNOG-Mapper as our BLAST-based approach led to similarly high levels of precision for the ciliate (0.996; supplementary table S7, Supplementary Material online) as we observed with TIdeS (0.987); however, EggNOG did not return annotations for 3,036 (∼40%) of the mixed kinetoplastid and *P. macrostoma* ORFs analyzed in the dataset.

**Table 3:** TIdeS classifies sequences from empirical datasets with high accuracy

Target-taxon	Association (contaminant)	Precision	Recall	F1-score	Training set approach
*Durinskia baltica*	Kleptoplasty (Diatom)	0.992	0.968	0.980	Phylogeny
*Durinskia baltica*	Kleptoplasty (Diatom)	0.984	0.979	0.982	Composition
*Fabomonas tropica*	Predator–prey (Bacteria)	0.897	0.912	0.904	Phylogeny
*Fabomonas tropica*	Predator–prey (Bacteria)	0.779	0.950	0.856	TIdeS + Kraken-8G
*Fabomonas tropica*	Predator–prey (Bacteria)	0.782	0.935	0.852	TIdeS + Kraken-16G
*Fabomonas tropica*	Predator–prey (Bacteria)	0.915	0.931	0.922	TIdeS + Kraken-Full
*Fabomonas tropica*	Predator–prey (Bacteria)	0.875	0.806	0.838	Kraken-Full
*Gleochaete wittrockiana*	Co-cultured (Amoebozoa)	0.953	0.976	0.965	Phylogeny
*Phytophthora infestans*	Host–pathogen (Tomato)	0.960	0.984	0.972	Phylogeny
*Phytophthora infestans*	Host–pathogen (Tomato)	0.975	0.969	0.972	Composition
*Platyophrya macrostoma*	Co-cultured (Kinetoplastid)	0.987	0.986	0.987	Phylogeny
*Telonema* sp.	Predator–prey (*Mantamonas*)	0.984	0.982	0.983	Transcriptomes

TIdeS accurately classifies ORFs from diverse types of common sources of “contamination”. Approach for generating training data for TIdeS (i.e. ORF composition, phylogenetic relationships, shared transcripts, and Kraken2) does impact the overall classification qualities, particularly in the identification of prokaryotic contaminants with the sequence classifier, Kraken2 (Wood et al. 2019). Combining TIdeS with the outputs of more “complete” Kraken2 databases greatly improves prediction quality in highly contaminated datasets.

We also identified evidence for eukaryotic cross contamination in the transcriptome of the glaucophyte alga *Gloeochaete wittrockiana* (MMETSP0308; SRX551388). Indeed, we observed a co-cultured amoeboid protist in the sequenced *G. wittrockiana* strain SAG 46.84 by light microscopy. From our set of diagnostic trees, we identified a substantial amount of ORFs related to discosid amoebozoans. Again, using a small selection of classified sequences (100 “glaucophyte” and 100 “amoebozoan” sequences), we quickly and accurately classified ∼35% (9,571/27,452) of the glaucophyte and amoebozoan ORFs (F1-score: 0.965; Table 3) across 4,210 phylogenetic trees. Ultimately, of the total 27,452 ORFs, we identified 7,109 (25.9%) ORFs belonging to the contaminant amoebozoan, providing a substantial pool of ORFs for further phylogenomic description. These results contrast substantially with ORF classification by eggNOG-mapper, which failed to annotate significant fractions of the glaucophyte and amoebozoan ORFs, performing significantly worse than TIdeS (F1-score: 0.328; supplementary table S7, Supplementary Material online).

Similarly, our initial survey of diagnostic phylogenies including *Fabomonas tropica* identified strong prokaryotic contamination. Given that *F. tropica* is a member of the deeply diverging eukaryotic clade of ancyromonads (Yubuki et al. 2023), prokaryotic contamination could strongly impact their placement in the eukaryotic tree of life and impact efforts to root the eukaryotic tree. Screening the reads from the *Fabomonas* transcriptome for prokaryotic contamination with Kraken2 and its “full” database, inferred rampant contamination, with ∼22.6% of the reads likely belonging to *Fabomonas.* This substantial contamination was also found in our selection of diagnostic trees, as ∼40% (309/785) of the predicted *F. tropica* ORFs were clear cases of prokaryotic contamination. In addition to selecting training sequences from the prokaryotic and eukaryotic pools from the diagnostic trees, TIdeS was run using Kraken2 and its preformatted databases (8 Gb, 16 Gb, and “full”) to assess TIdeS's suitability to automatically identify and classify noneukaryotic “contamination” from samples where phylogenetic affinities remain poorly resolved. For all approaches, TIdeS performed well, with training data selected from the “full” Kraken2 database being the best performing (F1-score: 0.922; Table 3), whereas the phylogeny-based and remaining Kraken-based approaches performed less well overall (Table 3). Simple filtering of the predicted ORFs with Kraken2's full database had reasonable precision and accuracy, although its overall performed was worse than the TideS-based approaches. Similarly, eggNOG-mapper showed reasonable results (F1-score: 0.875; supplementary table S7, Supplementary Material online), comparable to the best performing TideS-based approaches, although this requires considerable local resources or waiting times through the eggNOG API. While TIdeS can reduce prokaryotic contamination post-assembly, we also assessed the impact of Kraken2-based filtering of reads, prior to assembly. This preassembly quality measure reduced the representation of bacterial contamination by >90% in the assembled transcriptome (116/1,341 prokaryotic ORFs). Given Kraken2's efficacy for identifying prokaryotic and human contamination prior to assembly, we strongly recommend this as a primary means to reduce prokaryotic/human contamination, with numerable post-assembly (e.g. TIdeS, eggNOG-mapper) approaches performing similarly to further reduce these contaminants.

### Delineating Data in Organismal Associations

#### Host–Symbiont Interaction: Protist–Protist Endosymbiosis

The interconnectedness among some organismal associations is particularly difficult to tease apart in the absence of quality genomic representation. Dinotoms, dinoflagellates with diatom symbionts, are an interesting case of on-going symbiosis where the diatom symbiont remains fully intact rather than just its plastid in the dinoflagellate body (Yamada et al. 2019). To dissect and classify predicted ORFs from the dinotom *Durinskia baltica* and its diatom symbiont, we compared the performance of training set selection based upon phylogenetic tree-walking versus sequence composition (supplementary fig. S1, Supplementary Material online), noting that there are two very distinct ORF compositions present in its transcriptome. Training TIdeS using 100 sequences randomly chosen from each centroid (sequence-composition based) or from phylogenetic relationships (e.g. sequences sister to other dinoflagellates vs. diatoms; tree-walking based) was sufficient in discerning *Durinskia* from its endosymbiont, with composition-based approaches performing negligibly better than tree-walking approaches (composition F1-score: 0.982; tree-walking F1-score: 0.980; Table 2), as estimated from 10,549 classified sequences across 3,441 single-gene phylogenies. The paucity of publicly available dinoflagellate datasets was evident in our eggNOG-based ORF classification, resulting in poor overall performance (F1-score: 0.609; supplementary table S7, Supplementary Material online), with many misclassified ORFs attributed to diatoms.

#### Predator–Prey Interaction: Eukaryovory

Many established protist cultures rely on co-cultured prey organisms, which often include an assemblage of unknown bacteria (Aponte et al. 2021) and/or other protists (e.g. *Chlamydomonas reinhardtii*). For example, our established culture of the marine eukaryovore flagellate *Telonema* sp., is maintained by the prey protist, *Mantamonas sphyraenae*. Despite starvation prior to RNA-isolation, it was not possible to entirely dissociate *Telonema* from its prey. Indeed, >58.6% of *Telonema*'s RNA-seq reads were mapped to the currently available *M. sphyraenae* genome (Blaz et al. 2023), indicating substantial contamination from the prey organism in RNA-seq data. For many established cultures, genomes of the prey organisms are unavailable, so *Mantamonas*’ publicly available transcriptome was used in lieu of its genome to represent more “typical” lab conditions. Our training set was generated by comparing the TIdeS-predicted ORFs from the transcriptomes of *Telonema* sp. and *M. sphyraenae*, marking *Telonema*'s ORFs as either truly from *Telonema* or its prey (see Materials and Methods). This comparative transcriptome approach provided a strong basis for ORF classification (F1-score: 0.983; Table 3). This illustrates an alternative means to establish strong training sets in lieu of composition-informed or (rather time consuming) phylogeny-based training sets. This particular example is increasingly representative of efforts to describe eukaryotic diversity, as new major lineages of eukaryotes are described with relative frequency (Lax et al. 2018; Tikhonenkov et al. 2022). For BLAST-based approaches, these “orphan” and early diverging eukaryotic taxa represent unique challenges, as these taxa and their relatives often lack representation across established databases (e.g. RefSeq and eggNOG). This is evidenced by eggNOG-mapper's failing to produce any distinct hits attributable to either taxon (F1-score: 0.000; supplementary table S7, Supplementary Material online).

#### Host–Pathogen Interaction: Plant–Protist Parasitism

Most of our applications of TIdeS have been in highly contaminated datasets, often with relatively “balanced” numbers of ORFs between the target taxon and its contaminants/symbionts. To further evaluate TIdeS, we used data from an oomycete infection of a tomato plant. Despite high quality available whole genome datasets for both the pathogen *Phytophthora infestans* and the host plant *Solanum lycopersicum*, we evaluated the performance of TIdeS, generating training datasets using distributions of sequence composition (supplementary fig. S1, Supplementary Material online) and phylogenomic tree-walking approaches, as well as with eggNOG-mapper to delineate between the phytopathogen *Phytophthora* and its host. In this dataset, there is a strong imbalance in taxonomic representation in this dataset (>79.23% [12,289/15,510] of ORFs are from tomato), which is unsurprising, given the immense differences in taxon size and *Phytophthora*'s invasive and aggressive infection strategy (Leesutthiphonchai et al. 2018). Despite these imbalances, sequence classification with TIdeS was fairly accurate across both approaches for training set generation (composition F1-score: 0.972; tree-walking F1-score: 0.972) as well as with eggNOG-mapper (EggNOG F1-score: 0.992; Tables 3 and S7). EggNOG performed exceptionally well on this dataset because *Phytophthora*'s importance as a phytopathogen has led to strong representation of itself, related phytopathogens and various hosts, in numerous publicly available databases (e.g. RefSeq).

#### Running Time and RAM Requirements

We assessed the processing time and RAM requirements of GeneMarkS-T, Prodigal, TIdeS, and TransDecoder by running ORF predictions on each of the test datasets, to assess the overall computational requirements across a diverse sample of transcriptome features (e.g. low vs. high GC content, few vs. many transcripts, etc.), using a personal computer (2.6Ghz Intel core i7 and 16Gb RAM). All test datasets, were previously prepared with TIdeS to remove short transcripts, rRNA sequences, and redundant near-identical isoforms (i.e. the initial filtering stage performed by TIdeS). For TransDecoder, the run time and RAM requirements included the TransDecoder.LongOrfs and TransDecoder.Predict stages, as well as generating extrinsic hits from the UniProtKB/Swiss-Prot database. These tests demonstrated the rapid ORF predictions made by GeneMarkS-T and Prodigal (on average 18.23 s and 22.66 s, respectively), along with relatively low RAM requirements as well across our test transcriptomes (534.76 Mb and 538.65 Mb, for GeneMarkS-T and Prodigal, respectively; supplementary table S8, Supplementary Material online). By contrast, both TIdeS and TransDecoder run times were notable slower on average (94.38 s and 255.04 s, respectively), with greater RAM requirements as well (783.96 Mb and 861.11 Mb for TIdeS and TransDecoder, respectively). We do note that unlike TransDecoder, TIdeS's run time performance is improved through multithreading, requiring ∼38 s when using eight threads. From these results ORF predictions by TIdeS are ∼2× faster than TransDecoder using a single thread (∼6× faster with 8 CPU threads), whereas GeneMarkS-T and Prodigal are ∼14× and ∼11× faster than TransDecoder, respectively (supplementary table S8, Supplementary Material online).

We also evaluated the run time and RAM requirements by TIdeS for the empirical decontamination scenarios used in this work, excluding those relying on Kraken2, as the overall performance is largely a reflection of Kraken2's performance, rather than TIdeS. Deploying a single CPU thread, model training, and subsequent ORF classification by TIdeS takes ∼7.24 s on average with modest RAM requirements (∼440.38 Mb). Notably, using a pretrained model for ORF classification is slightly quicker, ∼5.3 s, with similar RAM usage (∼437.32 Mb) as performing a full training and subsequent classification with TIdeS.

## Discussion

With the rapid generation of high throughput sequencing data and the challenges with establishing pure (axenic) protist cultures, there is an increasing need for rapid approaches to delineate and clean data that are derived from both target and nontarget species. Data curation is nontrivial and typically time consuming, particularly in phylogenomic studies where, prior to the concatenated analyses, hundreds to thousands of individual gene phylogenies are often curated by eye to identify putative contaminant sources (e.g. from organelles, food sources, co-cultured organisms, and pathogens) common in studies of nonmodel taxa (Strassert et al. 2019; Schön et al. 2021; Cote-L’Heureux et al. 2022). To address these challenges, which include ORF identification and decontamination, we have developed a machine-learning tool, TIdeS, to ease the burden of these hurdles from limited datasets. By default, TIdeS generates “bespoke” models (ORF identification or decontamination) for a given dataset. As limited human curation is required for accurate model training by TIdeS (particularly for decontamination; Figs. 3 and 4), TIdeS can improve curatorial reproducibility and reduce burdensome human curation. As previously trained models can be easily shared and reused, ORF predictions and classifications can be quickly performed in the absence of compounding unconscious human biases made during manual curatorial approaches. This is particularly important for organisms where taxonomic affinities remain unclear, as unconscious biases (e.g. sequence exclusion given uncertain positions, inadvertent sequence selection based on presupposed hypotheses, inclusion/exclusion based on phylogenetic branch lengths, or mistaking inter-domain lateral gene transfers as contaminants and vice versa) can inadvertently impact our interpretations of evolutionary histories, organismal associations, and organismal biology.

For many studies lacking access to high-quality genome assemblies, de novo ORF prediction is the initial step following transcriptome assembly. Despite the prevalence of numerous ORF prediction software tools [e.g. CodAn (Nachtigall et al. 2021), Borf (Signal and Kahlke 2021), GeneMarkS-T (Tang et al. 2015), Prodigal (Hyatt et al. 2010), and TransDecoder (Haas et al. 2013)], TransDecoder remains the preeminent choice. Across 32 diverse eukaryotic taxa, encompassing microbial and multicellular eukaryotes, TIdeS-predicted ORFs with similar precision as well-established tools (GeneMarkS-T, Prodigal, and TransDecoder) and ultimately performs comparably. Overall, TIdeS performs similarly to GeneMarkS-T, the most robust of the tools tested general ORF prediction (Table 1). Notably, TIdeS and GeneMarkS-T strongly outperform the “standard” TransDecoder pipeline, which generally underperformed across the diverse eukaryotic dataset tested (Table 1). While established tools do natively predict *more* ORFs than TIdeS at similar precision, these predictions often comprise far fewer full-length ORFs than TIdeS (Tables 1 and 2; supplementary table S2, Supplementary Material online). Given the near “universal” use of TransDecoder for ORF prediction from eukaryotic transcriptomes, we compared TransDecoder and TIdeS's ability to identify full-length ORFs. Despite predicting more ORFs, default runs of TransDecoder predicted ∼25% fewer full-length ORFs (possessing start and stop codons with near perfect alignments to the respective proteome) in the CRF than TIdeS (supplementary table S2, Supplementary Material online). Notably, the full-length complete pORFs predicted by TIdeS cover a larger proportion of a taxon's proteome compared to the other tools tested (Table 2; supplementary table S2, Supplementary Material online) as well, although we note that the “longest-ORF” approach captures the greatest proteome breadth. Inaccuracies in ORF prediction can have severe impacts in our interpretations of organismal and genome evolution in studies relying upon transcriptome-based datasets. Whereas TransDecoder evaluates ORF log-likelihoods to identify the best putative ORF and can incorporate extrinsic information (“BLAST” hits against users’ preferred databases), TIdeS uses a (customizable) reference proteome database in ORF predictions. For our study, using a reference proteome database composed of data from six diverse eukaryotes, TIdeS still outperformed TransDecoder (as well as Prodigal and GeneMarkS-T) in correctly calling full-length ORFs (Fig. 2; Tables 1 and 2). However, we note that proteome databases from more closely related taxa (when available) may improve overall ORF prediction performance.

TIdeS can be particularly pertinent when processing eukaryote transcriptome data that inadvertently include nontarget reads. Typically, sequence curation is performed manually, incorporating information from phylogenetic trees, composition, “BLAST”-based approaches, or a combination thereof (Laetsch and Blaxter 2017). In our evaluation of BLAST-based approaches, there is a large discrepancy in its power to distinguish among contaminants. This largely reflects the current state of data availability from diverse lineages across the tree of life, as the relatively strong representation of prokaryotes, well-studied eukaryotic multicellular lineages (e.g. flowering plants, fungi, animals) and pathogens/parasites (e.g. the plant pathogen *Phytophthora*), provide a means for accurate and easily interpretable sequence classifications (supplementary table S7, Supplementary Material online). However, our analyses show the unsuitability of these approaches when evaluating contamination from poorly represented eukaryotic lineages. For these taxa, BLAST-based performances vary wildly (supplementary table S7, Supplementary Material online), ultimately completely failing to classify any ORFs in our highly contaminated dataset from the enigmatic protist *Telonema*. With the increased interest and description of novel *deep* lineages of eukaryotes (e.g. Provora; Tikhonenkov et al. 2022), TIdeS provides a robust means to accurately classify ORFs from these datasets. Given the link between “BLAST”-based performance and genomic representation, these approaches will improve with the generate of high-quality genomes from nonmodel microbial eukaryotes, although this supposes the genomes are free of severe contamination, which currently is not always the case even among well represented eukaryotic clades (e.g. metazoa).

Alternatively, phylogenetic approaches can circumvent the issues that homology-based searches encounter when taxa are from poorly represented areas of the tree of life. However, phylogenetic approaches can be less reproducible as they tend to rely heavily on human curation and when curatorial “rules” are not explicitly clear. We have shown that TIdeS can accurately classify sequences from binary to multiple classes with high precision. From our simulated and empirical datasets, we demonstrate multiple approaches for initial sequence classification (composition-based vs. phylogenetically informed) that are sufficient for fairly accurate ORF classification. As with all machine-learning based approaches, there are notable limitations that users ought to consider, including but not limited to: (i) the quality of the dataset, which is critical for accurate classifications (“garbage in, garbage out”), (ii) still relies on (reduced) human curation for sequence classifications, although TIdeS backs up these tables by default, and (iii) sequence classification requires *enough* “contamination” to accurately classify sequences. Despite these limitations, we have shown that minimal human curation in the generation of the training set is often sufficient for reproducible high-quality ORF classification with TIdeS, with no manual human curation needed for ORF prediction, which again can be easily reproduced using a shareable pretrained TIdeS model.

By providing accurate ORF predictions and the means to classify sequences with high precision in the presence of contamination, TIdeS provides a robust foundation for numerous downstream applications. The most common-sense use of TIdeS is the removal of contaminant sequences for phylogenomic studies, which is critical for deeply diverging eukaryovores with uncertain phylogenetic placement (e.g. *Telonema*). Similarly, it can be difficult distinguishing lateral and endosymbiotic gene transfer events from read contamination from the co-cultured microorganisms or library preparation. With evidence for rapid sequence amelioration to the host genome (Cote-L’Heureux et al. 2022), TIdeS can enable the identification of putative gene transfer events and reduce the likelihood of false reporting. TIdeS's ability to accurately identify in-frame (and often full-length ORFs) provides a means to better infer putative “young” lineage-specific genes from transcriptome datasets, as approaches for inferring these genes from transcriptomic data vary (Wilhelmsson et al. 2017; Hu et al. 2020). Additionally, TIdeS's ability to accurately delineate between sequence classes, even in the presence of extreme dataset imbalances that are common in host–microbe interactions (e.g. *Phytophthora* infection of tomato), can enable more thorough investigations of organismal interactions, especially when it is challenging to dissociate the target taxon from the host material (e.g. host–pathogen). Overall, TIdeS provides an entry-point for many ‘omics-based studies providing accurate ORF identification and curation, especially from contaminated microbial eukaryote datasets.

## Materials and Methods

### TIdeS Model

For predicting ORFs with TIdeS, transcriptomes undergo several pre-preprocessing steps: rRNA identification with Barrnap (v0.9; Seemann 2024) and removal using each of Barrnap's modeled “kingdoms” (bacteria, archaea, mitochondrial, eukaryotic), followed by clustering to remove the overabundance of redundant isoforms (a common issue for some de novo assemblers such as Trinity; Grabherr et al. 2011) using CD-HIT-EST (v4.8.1; Fu et al. 2012): “cd-hit -G 0 -c 0.97 -aS 1.0 -aL 0.005”. We provide an additional option to remove “noneukaryotic” contamination using the metagenomic profiler Kraken2 (v2.1.3; Wood et al. 2019) and a user-supplied Kraken2-formatted database. Following sequence curation, filtered transcripts are then aligned to a reference proteome using DIAMOND (v2.2.0; Buchfink et al. 2021): “-e 1e-30 -k 1”. We provide the means to generate the same small proteome used as a utility script (available: github.com/xxmalcala/TIdeS/tree/main/util/prep_tides_db.sh). Aligned ORFs are captured and additionally converted into a random orientation (+2/+3 or −1/−2/−3), representing the pool of training ORFs. ORFs are predicted from all orientations of each transcript using a user-given genetic code. By default, only complete ORFs are identified from the transcripts, representing the query dataset.

For sequence classification, initial ORF classifications are either user provided, or if a Kraken2 database is provided, determined from the suite of noneukaryotic classifications generated by Kraken2 (with 25–100 sequences from each category: “eukaryotic,” “bacterial,” “archaeal,” and “viruses”). These classified sequences, either user-defined or Kraken2-defined, serve as the training data, whereas the remaining sequences are the query dataset.

Following training-query data preparation, codon compositions for both datasets are tokenized with SciKit-Learn's CountVectorizer. Afterwards, tokenized counts of the training ORFs are used for supervised training of a support vector classifier (SVC) as implemented in SciKit-Learn (v1.5.0; Pedregosa et al. 2012). Optuna (v3.4.0; Akiba et al. 2019) was used to tune the SVC: “probability’: “True,” “kernel”: “rbf,” “C”: [1e−8, 10] with 5-fold cross validation. SVC probabilities were used to determine the best classification for each ORF. For ORF prediction when ORFs with identical probabilities of being in-frame are found, TIdeS defaults to the larger ORF whenever possible.

### Data Collection, Processing, and Dataset Creation

Accession information for the raw reads and proteomes from representative diverse eukaryotic taxa can be found in supplementary table S1, Supplementary Material online. Raw RNA-seq reads were trimmed and filtered using bbduk.sh from the bbtools package (2014) using: “ktrim = r k = 21 mink = 11 hdist = 1 qtrim = rl trimq = 24.” Trimmed RNA-seq reads were then assembled de novo using SPAdes (v3.15; Bushmanova et al. 2019) with the “–rna” flag.

Of the taxa in this study, we selected 32 taxa with quality genome annotations to capture the breadth of eukaryotic diversity to evaluate ORF predictions. To determine the TIdeS's ability to classify sequences (e.g. host vs. pathogen, contaminant vs. target), we identified additional six datasets based on their BioProject descriptions (e.g. oomycete infection of tomato), organismal biology (e.g. kleptoplasty), and presence of possible contamination (see Phylogenomic Curation of Transcriptome Datasets). In addition to these empirical datasets, we generated an in silico “contaminated” dataset from taxa with whole genome representation by pooling trimmed reads from the predatory freshwater ciliate *Stentor coeruleus* and two “prey” algae, *Phaeodactylum tricornutum* and *Chlamydomonas reinhardtii*, prior to assembly and 2) combining proteomes from representative taxa in differing proportions (supplementary table S5, Supplementary Material online). While *Phaeodactylum* is a marine diatom, of the few diatom genomes available, we chose *Phaeaodctylum* as it possesses one of the highest quality genomes among publicly available diatom genomes. We identified three clusters based on the composition of GC content at 1st/2nd codon positions (GC12) and 3rd positions (GC3) and selected a random subset of 100 sequences delineating among clusters as follows: Cluster-1: 35% to 45% GC12 and 25% to 35% GC3, Cluster-2: 45% to 55% GC12 and 45% to 55% GC3, Cluster-3: 45% to 70% GC12 and 80% to 100% GC3.

### ORF Prediction

Following transcriptome assembly, TIdeS was used to predict ORFs under default parameters for all taxa, with the exception of the ciliate, *Tetrahymena thermophila*, where the “ciliate” genetic code was provided. Given the variability in machine-learning predictions and training set construction, all runs of TIdeS were performed in triplicate and with default parameters, including the option of excluding partial ORFs. The initial filtering provided by TIdeS included size filtering, rRNA removal, and initial isoform clustering. After this, ORF prediction was made with popular tools (TransDecoder, GeneMarkS-T, and Prodigal), selecting the longest complete ORFs (i.e. possessing start and appropriate stop codons) to ensure similar comparisons. For ORF predictions with TransDecoder (v5.5.0; Haas et al. 2013), initial ORF predictions were generated using TransDecoder.LongORFs with default parameters (using “–genetic_code Tetrahymena” for *Tetrahymena thermophila*). TransDecoder's “longest_orfs.pep” data were then used to captured extrinsic information using DIAMOND (v2.2.0; Buchfink et al. 2021) for BLASTP searches against the UniprotKB/Swiss-Prot database (last accessed July 2024), using the DIAMOND options: “–very-sensitive -k 1 -e 1e-5”. TransDecoder.Predict was used to generate TransDecoder's final ORF predictions, incorporating the output from the homology searches against UniProtKB/Swiss-Prot. Predictions using GeneMarkS-T (v5.1.0; Tang et al. 2015) were performed using default options. We note that GeneMarkS-T does not support alternative genetic codes, and so was not used for ORF predictions for *Tetrahymena thermophila*. Finally, ORF predictions using Prodigal (v2.6.3; Hyatt et al. 2010) were performed using a full-motif scan (“-n”) and using the appropriate genetic code (e.g. universal: “-g 1” or “ciliate”: “-g 6”).

### Putative ORF Evaluation and Validation

DIAMOND (v2.2.0; Buchfink et al. 2021) was used to determine the proportion of predicted ORFs in the CRF in comparisons with the appropriate reference proteome (script is provided “ref_compare.py”): “blastx -k 1 –very-sensitive -f 6 qseqid sseqid qlen slen length pident qframe.” As an additional level of stringency, we also looked at the proportions of “full” ORF predictions, appending “-query-cover 98 –subject-cover 98” to the same command. For evaluating partial ORFs, we define phylogenomically “useful” partial ORFs as those aligning to at least 66% of the reference protein's length, appending “-query-cover 98 –subject-cover 66” to the base DIAMOND command. To capture the maximal number of CRF ORFs, all ORFs were predicted from the processed transcriptomes and evaluated with the same DIAMOND command/script.

As evaluation metrics, we used the *precision* (Precision = TP/(TP + FP))—the percentage of pORFs found in the CRF, the *recall* (Recall = TP/(TP + FN))—the percentage of pORFs in the CRF from the total possible pool, and the F1-score—the harmonic mean of precision and recall. For all tools and approaches, evaluations were based on the total ORF predictions under default parameters, which includes partial ORFs generated by TransDecoder, GeneMarkS-T, and Prodigal, by default. Complete and partial ORFs were predicted separately with TIdeS, which provides searches for complete ORFs by default and partial ORFs using the “-p” option.

### Assessment of Transcriptome Quality

Following ORF calling with TIdeS, we analyzed sequence codon-based composition metrics including G + C content at third-positions (GC3), at third position 4-fold degenerate sites (GC3 s), and at first and second sites (GC12) using a custom python script (orf_composition.py) provided as a utility script: github.com/xxmalcala/TIdeS/tree/main/util/orf_composition.py. Composition metrics can represent taxonomic “fingerprints” and have been used to aid in identifying putative contamination prior to generation of phylogenomic evidence (Cote-L’Heureux et al. 2022). We used these metrics to confirm strong signatures of contamination in our empirical sequence-classification datasets, and to create simulated datasets that resembled our empirical datasets (e.g. with varying degrees of sequence composition similarity). We also provide an additional python script (seqs_by_composition.py) that uses the outputs of the “orf_composition.py” along with user-inputs to randomly select sequences meeting composition thresholds (e.g. GC12 between 20% and 30% as well as GC3 between 70% and 85%). Additionally, for assessing the degree of prokaryotic contamination in the *Fabomonas* dataset, we curated trimmed reads with Kraken2 and the “full” Kraken2 database, using the “–quick” option.

### Phylogenetic Curation of Transcriptome Datasets

For the empirical sequence classification datasets, PhyloToL (Cerón-Romero et al. 2019) was used to assign TIdeS-predicted partial ORFs to widely conserved eukaryotic gene families and generated initial taxon-rich multisequence alignments with MAFFT (v7.490; Katoh and Standley 2013). We selected 5,643 gene families, which included data from at least three of the contaminated taxon datasets and include 408 gene families that have been used for the general assessment of data quality (Cote-L’Heureux et al. 2022). While PhyloToL does include the means to further curate and remove nonhomologous sequences from the multisequence alignments (MSAs), we chose to skip this additional curation step given our conservative criterion for phylogeny-based sequence classification (described below). MSAs were then trimmed with ClipKit (v1.4.0; Steenwyk et al. 2020) using the “smart-gap-kpic” model. Individual gene trees were constructed using FastTree2 (v2.2.2.6; Minh et al. 2020) with the “-lg -gamma” flags.

Following gene tree construction, we curated every sequence in the gene trees, focused on the contaminated taxa, and binning into three broad categories: (i) sequences sister to nonclosely related taxa (e.g. sister to known prey taxa) along short branches (branches shorter than the median branch lengths) to mitigate possible long branch attraction artifacts—marked as nontarget sequences, (ii) sequences sister to other related taxa where representation is strong—marked as target sequences, and (iii) sequences with no clear phylogenetic affiliation—marked as noninformative.

### Training Data Selection

For simulated datasets where we combined protein-coding CDSs from different taxa, we randomly selected 25, 100, and 200 sequences from each taxon as our training datasets. For the multiclassification example, a random set of 100 sequences were selected from each of the three densest regions in the composition plots for training TIdeS.

For *Durinskia baltica* CSIRO CS-38*, Fabomonas tropica* NYK3c, *Gloeochaete wittrockiana* SAG 46.84, *Phytophthora infestans* isolate 88069, *and Platyophrya macrostoma* WH, 100 random sequences were chosen based on the phylogenetic placement of putative target sequences in large clades of other closely related taxa (same “minor clade”; given the uncertain phylogenomic placement of *Fabomonas*, “eukaryotes” were used as the “minor clade’) or robust contaminant (nontarget) clades from the diagnostic set of phylogenies. Additionally, we evaluated the use of sequence composition in generating training data for assessing host and symbiont in the dinoflagellate *Durinskia baltica* as well as for assessing host and pathogen in a *Phytophthora infestans* infection of tomato. For *Durinskia*, two clusters were identified based on their composition (cluster 1: 45% < GC12 < 55% and 35% < GC3 < 55%; cluster 2: 45% < GC12 < 60% and 75% < GC3 < 100%). For *Phytophthora*, we again identified two apparent clusters based on their composition (cluster 1: 45% < GC12 < 55% and 35% < GC3 < 50%; cluster 2: 45% < GC12 < 55% and 55% < GC3 < 75%). Additional training sequences for *Fabomonas* were selected automatically using Kraken2 (Wood et al. 2019) as implemented by TIdeS, using the preformatted 8 Gb, 16 Gb, and full Kraken2 databases to assess Kraken2's utility in “automated” curation of noneukaryotic sequences.

As the culture of the eukaryovore, *Telonema* sp., relies on *Mantamonas sphyraenae* as a prey organism, the de novo assemblies and TIdeS ORF predictions for both *Telonema* sp. and *M. sphyraenae* were prepared using TIdeS's default parameters. HISAT2 (Kim et al. 2019) was used to map *Telonema*'s raw transcriptome reads against the available *M. sphyraenae* genome (Blaz et al. 2023) to confirm substantive contamination of its prey. To develop a training dataset, the predicted ORFs for *Telonema* sp. and *M. sphyraenae* were clustered together using CD-HIT-EST (Fu et al. 2012) using “-G 0 -c 0.97 -aS 1.0 -aL 0.0005.” Clusters containing *Telonema* sp. and *M. sphyraenae* together were considered evidence of the prey contamination, whereas clusters containing solely *Telonema* sp. were considered “clean,” with 100 sequences annotated from each category as the training set.

### Supervised Sequence Classification and Validation

For all datasets, TIdeS was run using “-c [file-name]” where “file-name” represents the respective table of annotated sequences. For *Fabomonas*, TideS was run using “-c -k [kraken2-database]” using the preformatted 8 Gb, 16 Gb, or full databases to infer the utility of the preformatted Kraken2 datasets for handling noneukaryotic contamination. All annotated training sequences are excluded from our analyses of TIdeS's performance metrics. Calculations of precision, recall, micro-F1-score for the empirical datasets were performed using the annotated sequences present in the set of 7,289 single-gene phylogenies (excluding *Telonema*), whereas calculations for simulated datasets were based on the known taxonomic sources for all sequences. Classified sequences for *Telonema* were scored by using BLASTN (Camacho et al. 2009) to map classified ORFs against the *Mantamonas* genome at 95% identity. The resulting “presence–absence” data were then used for all scoring metrics.

### ORF Classification With eggNOG-Mapper and eggNOG-DB

To assess the putative taxonomic origins of the predicted ORFs for classification, we used the eggNOG-mapper tool (Cantalapiedra et al. 2021), using DIAMOND (Buchfink et al. 2021) with default parameters. Taxonomic identities were assigned simply based on the highest bit scores *per* sequence while accounting for known sources of contamination. The categories we used for accepting the eggNOG-mapper outputs varied by taxon and its contaminant: *Lithophyllum* (any sequences marked as “Viridiplantae”), its metazoan and rhizarian contaminants (any sequences marked “Metazoa” and other “Eukaryota,” respectively), *Durinskia baltica* (any sequences marked as “Eukaryota,” but *not* “Bacillariophyta”) and its diatom contaminant (any sequences annotated as “Bacillariophyta”), *Fabomonas tropica* (any sequences marked as “Eukaryota”) and its prokaryotic contaminants (any sequences marked as “Bacteria” or “Archaea”), *Gloeochaete wittrockiana* (any sequences marked as “Viridiplantae”) and its amoebozoan contaminant (any sequences marked as “Amoebozoa”), *Phytophthora infestans* (any sequences marked as “Peronosporales”) and its tomato host (any sequences marked as “Viridiplantae”), *Platyophrya macrostoma* (any sequences marked as “Ciliophora”) and its kinetoplastid contaminant (any sequences marked as “Kinetoplastida”), and *Telonema* and its prey *Mantamonas sphyraenae* were indistinguishable through eggNOG (both returning hits to a seemingly random assortment of taxa).

## Supplementary Material

evae252_Supplementary_Data

## Data Availability

The TIdeS source code and installation instructions are available at: https://github.com/xxmalcala/TIdeS. A compressed archive of the source code, as well as the data generated in this study are available at the Zenodo research data archive at: 10.5281/zenodo.10927824.
