# Supplementary material for: TIdeS: A Comprehensive Framework for Accurate Open Reading Frame Identification and Classification in Eukaryotic Transcriptomes
Source: Genome Biol Evol. 2024 Nov 21;16(12):evae252. doi: 10.1093/gbe/evae252 (PMC11631190; doi:10.1093/gbe/evae252)
Supplement: evae252_Supplementary_Data [file evae252_supplementary_data.zip › XXMA_TIdeS_Revised.SuppFigTable_Info.docx]

Figure S1. Composition plots of emperical contaminated datasets. Plots of GC content in first and second codon positions (y-axis) and GC content of third codon positions (x-axis) for the dinoflagellate, *Durinskia baltica*, and its diatom endobiont, as well as the plant pathogen *Phytophthora infestans* and its tomato host.

Table S1. Accession information for the taxa used in this work. We note whether each taxon was used for evaluating TIdeS’s ORF-prediction and/or ORF-classification (e.g., contamination) workflows.

Table S2. ORF-prediction summary for all approaches evaluated in this work, which includes evaluating performance for complete full-length ORFs and partial ORFs.

Table S3. ORF-classification summary statistics for binary *in silico* contamination.

Table S4. TIdeS performance for *in silico* multi-classification dataset with one target taxon and two contaminants.

Table S5. Proportion of reads from each source that was pooled prior to *de novo* assembly for making an *in silico* multi-contaminant transcriptome.

Table S6. Performance metrics for TIdeS and eggNOG-mapper in classifying ORFs from an emperical red-algal transcriptome with two distinct contaminants (rhizarian and unknown metazoan).

Table S7. Summary of eggNOG-mapper’s decontamination performance for the emperical contaminated datasets.

Table S8. Overview of CPU and RAM requirements for ORF-prediction for TIdeS, Prodigal, GeneMarkS-T, and TransDecoder. Data provided include running TIdeS with one thread and eight threads, whereas all other approaches were run as described in the text.
