# Supplementary figures and images for: TIdeS: A Comprehensive Framework for Accurate Open Reading Frame Identification and Classification in Eukaryotic Transcriptomes
Source: Genome Biol Evol. 2024 Nov 21;16(12):evae252. doi: 10.1093/gbe/evae252 (PMC11631190; doi:10.1093/gbe/evae252)

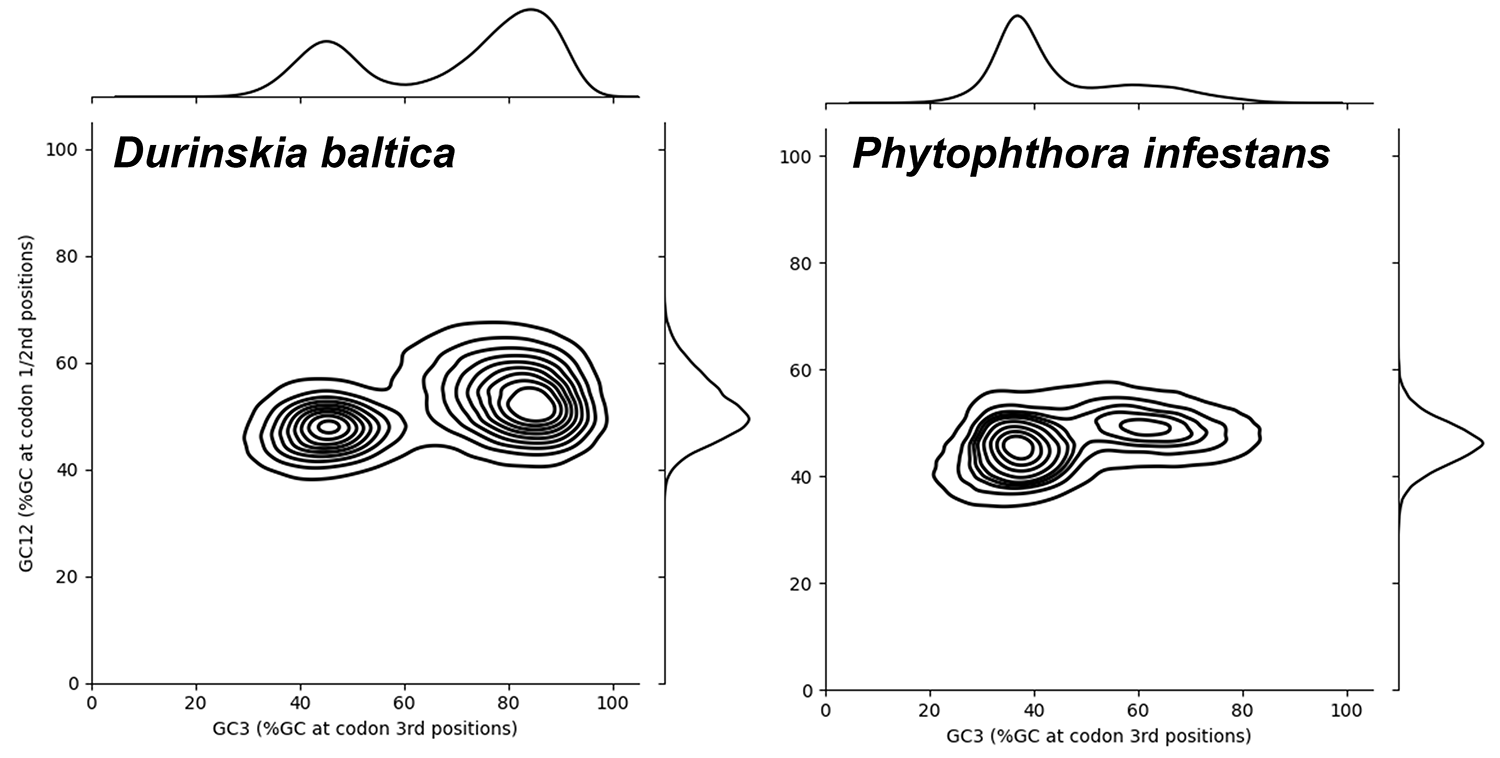

Supplement: evae252_Supplementary_Data [file evae252_supplementary_data.zip › Fig_S1.tif]
